# The Relative Carcinogenic Activity of Six Isomeric Aminoazotoluenes

**DOI:** 10.1038/bjc.1949.43

**Published:** 1949-09

**Authors:** H. G. Crabtree


					
387

THE RELATIVE CARCINOGENIC ACTIVITY OF SIX ISOMERIC

AMINOAZOTOLUENES.

H. G. CRABTREE.

From. the Laboratories of the Imperial Cancer Research Fund, London, N. W.7.

Received for publication August 22, 1949.

IT has been suggested from studies of the action of inhibitors of S-metabolism
on tumour induction by carcinogenic hydrocarbons that S-metabolism and the
process of carcinogenesis are closely linked (Crabtree, 1947). This association is
emphasized when the properties of azo-carcinogens are considered. The products
of their metabolism have been shown to inhibit the activities of urease (Potter,
1942) and succinoxidase (Elson and Hoch-Ligeti, 1944), two enzymes dependent
upon intact SH-groups for their proper functioning. Kensler, Dexter and Rhoads
(1942) studied the relative inhibitory action of a series of " split-products " on a
standard diphospho-pyridine-nucleotide system, and sought to correlate this
activity with the carcinogenic power of the parent azo-compound. Suggestive
parallelisms were found, but a clear-cut relationship did not emerge.

Much of this work has been based on the conception (experimentally verified
for p-dimethylaminoazobenzene by Stevenson, Dobriner and Rhoads (1942),
and for azo-benzene by Elson and Warren (1944)) that an azo-carcinogen is
initially metabolized by the process of reductive fission with the liberation of
an amine and a diamine, and that the latter is the effective carcinogen by virtue
of its capacity to interfere with normal enzymic. activities.

It is not clear why the diamine moiety alone should have been conceived as
having special significance for the process of cancer induction. A survey of the
azo-compounds so far tested for carcinogenic activity makes it evident that in
some cases the amine moiety of the reduced molecule plays a significant role in
determining potency, e.g. on comparing p-dimethylaminoazobenzene with its
p'-methyl derivative, the introduction of the p'-methyl group causes a change
from a very strong to a very weak carcinogen.

The present work was undertaken to probe this problem further. Isomeric
aminoazotoluenes were chosen as suitable for a study of the relative contributions
made by the amines and diamines which would arise from their reduction. The
well-known carcinogen 4'-amino-2 3'-azotoluene (o: o) and the non-carcinogen
2'-amino-4: 5'-azotoluene (p: p) served as bases of reference, and their behaviour
was compared with that of the hybrid compounds o: p and p: o. Two other
isomers-i : m and p: m-were also tested to provide further comparative
data.

Note on abbreviatioms used.-All the isomeric aminoazotoluenes are prepared
by coupling diazotized o-, m-, or p-toluidine with o-, m-, or p-toluidine, and the
abbreviations reflect their molecular structure, e.g. p: m signifies that diazotized
p-toluidine is coupled with m-toluidine, forming 4'-amino-4: 2'-azotoluene, and
so on.

H. G. CRABTREE

MATEBIAL AND METHODS.

Animals.-The experiments were carried out with rats of strain 52, descen-
dants of a group originally selected for high susceptibility to transplantation of
Jensen's rat sarcoma, and mice of the Simpson strain. A total of 210 rats
weighing from 80 to 110 g., and 280 mice, 3-4 months old, were used, each divided
into 7 groups of 30 rats or 40 mice. Six groups of each species were fed on the
basal diet supplemented by 0* 06 per cent of one of the six aminoazotoluenes, and
the seventh group, fed on the basal diet alone, acted as controls.

In order that the possible influence of sex could be observed, each group of
rats was divided into sub-groups of 10 males, 10 females, and 10 with members
of both sexes. A similar separation was made with the mice into corresponding
sub-groups of 10, 10 and 20.

Each cage or box contained 5 animals, and excess of the diets was always
present, together with water ad libitutm.

Diet.-The semi-synthetic diet was based on the one named R.D. 3 by Kirby
(1947) which, in turn, closely resembled that developed by Miller, Miner, Rusch
and Baumann (1941). as being suitable for promoting a high incidence of liver
tumours with p-dimethylaminoazobenzene and related compounds. The latter
was low in protein content, inadequate in some members of the B group of vita-
mins, and incapable of promoting normal growth in rats. The single source of
carbohydrate was glucose monohydrate, for which Kirby substituted starch in
his R.D. 3 diet. The carbohydrate used in the experiments described here was
commercial dextrin, with 5 per cent sucrose added to increase palatability.

Basal diet.                                          Per cent.

Casein (B.D.H. soluble, white, light)  .  .  .   12
Dextrin (B.D.H. technical yellow)  .   .    .   71
Sucrose  .    .    .    .    .    .    .    .    5
Salts (Harris, Krahl and Clowes, 1947)  .   .    4
Arachis oil   .    .    .    .    .    .    .    5
Cod-liver oil  .   .    .    .    .    .    .     1
Dried yeast   .    .    .    .    .    .    .    2

The dry constituents were mechanically mixed and the two oils then added with
further mixing. The azo-dye was dissolved in the oils in an amount representing
0 06 per cent of the complete diet.

N.B.-When 0 06 per cent of p-dimethylaminoazobenzene was incorporated
in this basal diet and fed to rats, liver tumours were produced in 90 per cent of
the animals during 3-5 months.

Isomers of amrinoazotoluene used.

Name of isomer.                   Abbreviation.
2'-amino-4: 5'-azotoluene.  .    .    .   p: p
4'-amino-4: 2'-azotoluene.  .    .    .   p: m
4'-amino-3 2'-azotoluene.   .    .    .  m: m
4'-amino-4: 3'-azotoluene.  .    .    .   p o
2'-amino-2: 5'-azotoluene.  .    .    .   o p
4'-amino-2: 3'-azotoluene.  .         .  .  o 0

388

CARCINOGENIC ACTIVITY OF AMINOAZOTOLUENES

Preparation of the aminoazotoluenes.

O: o.-Obtained commercially. Purified by crystallization from aqueous
EtOH. Long, red-brown prisms with blue sheen. M.P. 1000.

m: m.-A solution of 13-8 g. (1 mole) NaNO2 was added slowly to 57 4 g.
(2 moles) m-toluidine hydrochloride dissolved in 200 ml. water, at room tem-
perature (Mehner, 1902). The crystalline hydrochloride suspended in EtOH
was neutralized with ammonia, and an equal volume of boiling water added.
The free base was re-crystallized from aqueous EtOH. Golden yellow needles.
M.P. 810.

p: p (a) 4: 4'-diazoaminotoluene.-Prepared by a modification of the method
Gf Nietzki (1877). 96- 6 g. (2 moles) p-toluidine hydrochloride were dissolved in
350 ml. H20 and cooled to 5-7?. A solution of 23-4 g.(1 mole) of NaNo2 was
slowly run in over 2 hours with stirring. The pale yellow, microcrystalline
diazoamino-compound was used for stage (b) without further purification. M.P.
114-115 . Crystallization from aqueous EtOH gave large prisms. M.P. 1160.

(b) 2'-amino-4: 5'-azotoluene.-(p: p) 1 mole (a) + 1 mole p-toluidine hydro-
chloride + 6 moles p-toluidine were heated at 65? for 10 hours (Zincke and
Lawson, 1886). The molten mass was neutralized with dilute NaOH, p-toluidine
removed by steam distillation, and the brown residue crystallized from aqueous
* EtOH. Orange red needles. M.P. 1190.

p: m.-67* 5 g. 4 : 4'-diazoaminotoluene (1 mole) + 43 05 g. m-toluidine
hydrochloride (1 mole) + 450 ml. EtOH were kept at 20 for 3 days, with occasional
shaking (Mehner, 1902). The crystalline dye-hydrochloride was filtered (35 g.)
and a second crop (19 g.) was obtained after addition of 500 ml. N HCl-and standing
overnight at 20. Recrystallization from aqueous EtOH gave orange-red needles
with metallic sheen. The base was obtained as described for m: m. Large
rectangular golden-yellow plates. M.P. 128?.

p: o.-Prepared by method used for p: m (Mehner, 1902). After 5 days
at 2? the red-brown solution was treated with an equal volume of N HCI, and
EtOH largely removed on the water bath. The dye-hydrochloride crystallized
after standing overnight at 20. Yield = 66 per cent theory. The base was
obtained as described for m : m. Orange-red needles. M.P. 1280.

o: p (a) 2: 2'-diazoaminotolurene.-Based on the method of Fischer and
Wimmer (1887). 28 7 g. o-toluidine hydrochloride (2 moles) + 8 7 ml. HOC
(S.G. 1 18 - 1 mole) + 100 ml. H20 were cooled to - 50, and a solution of 6 9 g.
NaNO2 (1 mole) added during 5 minutes. A solution of 25 g. sodium acetate
was slowly run in, and, after stirring for 4 hours, the pale yellow, micro-crystalline
diazoamino-compound was collected. Yield = 70 per cent theory. M.P. 470.
This was used for (b) without further purification.

(b) 45 g. (a) (1 mole) + 28^ 7 g. p-toluidine hydrochloride (1 1 moles)
+ 380 ml. EtOH were kept at 20 overnight. 300 ml. N HCI were added and
EtOH largely removed on the water bath. Yield of dye hydrochloride was 47 g.
(90 per cent theory). Recrystallization from aqueous EtOH gave orange-red
needle clusters. The base was obtained as described for m: m. Orange-red
rectangular plates. M.P. 950.

EXPERIMENTAL RESULTS.

The livers, and sometimes other organs, of all animals were examined at
death, or whenever possible when death was inmnminent.

389

H. G. CRABTREE

Many rats were lost through cannibalism and intercurrent infection, princi-
pally endemic bronchopneumonia. Mice responded badly to the basal diet,
with or without the addition of dye. Many were lost in the first 6 months of the
experiment, but the death-rate was lowered in the later stages by feeding them
with standard laboratory food and the synthetic diet alternately for periods of
two weeks.

TABLE I.-Changes Produced by Six Isomeric Aminoazotoluenes in the Livers of

Rats and Mice, surviving 300-595 days. The histological classification is
based on a suwcession of changes reflecting increasing degrees of liver damage,
and each x refers to the liver of one animal.

MOUSE LIVERS

Under these circumstances quantitative estimates of the relative tumour-
inducing properties of the various aminoazotoluenes were not easy to make.
However, since the survival rates in the different groups of animals were not
widely different, a qualitative picture of the broad features of the results emerged,
and some significant differences in the action of the six isomeric compounds were
demonstrated.

(a) Histological observations.

Omitting animals which died before 250 days, the average time of survival
of both rats and mice in the various groups was 400-435 days from the beginning
of the experiment. A few were alive after 595 days and were killed.

The whole liver was removed, fixed in Bouin and sections made from several
or all of the lobes, and stained with eosin and haematoxylin. This was necessary,
since macroscopically visible tumours were rare.

I

I

390

CARCINOGENIC ACTIVITY OF AMINOAZOTOLUENES

The histological findings are collected in Table I, where each x refers to the
liver of one animal. The succession of changes used in this classification reflect
increasing degrees of liver damage. The first characteristic abnormalities visible
in the livers of rats differed from those commonly observed in the livers of mice,
though the final pictures of tumour emergence were similar in both species. In
building up the general picture four grades of cellular change have been used.

In rat livers perilobular necrosis constituted the primary change, and this
was followed by regeneration of liver cells in the necrotic areas. In some regions
of regeneration microscopic nodules of hepatoma made their appearance, and later
these became visible to the eye as dull grey patches on the surface of the lobes.
Often a simultaneous intense proliferation of bile ducts was manifest in one or
more lobes, and the early phases of cholangioma formation would coincide with
the development of small hepatomas. No metastases were ever found.

In mouse livers no preliminary necrosis was observed, but the cellular pattern
became abnormal with great irregularity in the disposition and size of the nuclei.
Further distortion was accompanied by the formation of cells with giant vacuo-
lated nuclei, which preceded the emergence of microscopic hepatomas. The
fully developed hepatomas of mice were more malignant than those of rats as
judged by the degree of atypical growth, though again no metastases were ever
found.

It will be noted that the earliest histological changes mentioned sometimes
occurred in both rats and mice consuming the basal diet without dye-addition.
This response of the liver to diets inadequate in several essential food factors may
well reflect the basic disturbance of liver function which is a necessary precursor
to the carcinogenic action of azo-compounds.

(b) Relative carcinogenic activity of the six isomeric amrnoazotoluene8.

Considering the data summarized in Table I from the point of view of the
relative carcinogenic potency of these isomers, three points emerge:

1. Mice were more susceptible than rats to the action of these azo-compounds.
With the exception of p : p, which proved innocuous in both species, all the isomers
were capable of inducing liver tumours in mice, but varied in potency among
themselves. In three cases, o : o, o : p and p : m, malignant hepatomas were
produced, and severe lesions, with one microscopic hepatoma, were caused by
m : m.

2. Only two isomers-o : p and o : o-induced tumours in rats. In all
other cases, except for one rat receiving p : o, perilobular necrosis represented
the utmost limit of damage, and this lesion was found, though in lesser degree,
in the livers of rats fed on the basic diet alone.

3. Except in the case of p : m, which induced a high percentage of tumours
in mice surviving 400 days and was innocuous for rats, the relative potency of
the isomers was similar in both species.

(c) Growth rate of rats on basal diet with azo-dye addition.

All rats (but not mice) were weighed at fortnightly intervals throughout the
experiment. Small differences of growth-rate within the various groups became
noticeable after 3-4 months, and later they became more accentuated. Since
all other environmental factors were similar for all the groups, these differential
growth rates could only be attributed to the azo-dyes themselves.

26

391

H. G. CRABTREE

The possible influence of sex on the growth rate was assessed by keeping the
rats of each series separated into three sub-groups containing respectively
10 males, 10 females and 5 males + 5 females. Though the average female rat
weighed 15-20 g. less than the average male of the same age, the differential
growth-rates due to the added azo-dyes were evident in both sexes, and were
unaffected by pregnancies, since breeding did not occur after the first few months
of the experimental period.

-   Weeks

FIG. I.-Growth curves of rats fed on the basal diet, with addition of 0106 per cent of an

aminoazotoluene. The differential effects on growth of the six isomers are shown.

The points shown in the curves in Fig. 1 are therefore based on the average
weights of each group of rats, regardless of sex. The following features of these
growth-curves may be emphasized:

1. The basal diet was inadequate for normal growth, but permitted limited
growth and long survival.

2. The two isomers o : o and o : p had no inhibitory action on body growth,
or more exactly, they had no additional effect on growth already inhibited by
the basal diet.

3. The four remaining isomers-p : p, p: m, m: m      and p : o-caused a
stimulation of growth-rate in varying degrees, the piost effective being the p : p
isomer.

I

I1

392

CARCINOGENIC ACTIVITY OF AMINOAZOTOLUENES

On comparing these effects on rat-growth with the histological data in Table I
a suggestive correlation is obvious; all the isomers which caused growth-stimu-
lation were non-carcinogenic, whereas those lacking this property were carcino-
genic, for rats.

(d) Growth rate of rats on basal diet, with addition of toluidin.es.

Two investigations have shown that azo-compounds are metabolized in the
rat by initial reduction at the azo-group, yielding the corresponding amines
(Stevenson, Dobriner and Rhoads, 1942; Elson and Warren, 1944). On the
assumption that this splitting process occurred with the aminoazotoluenes used

-     Weeks

FIG. 2.-Growth curves of rats fed on the basal diet, with addition of 0 - 06 per cent o- or p-toluidine.

in the work reported here, the two carcinogenic isomers would yield o-toluidine,
while 3 of the 4 growth-stimulating isomers would yield p-toluidine as the primary
products of reductive fission.

The possible effects on growth of these two toluidines were therefore tested.

Forty-five rats of the 52 strain, each weighing 110-130 g., were divided into
three groups of 15. Two groups were fed on the basal diet containing 0.06 per
cent of either o-toluidine or p-toluidine, and the third group received the basal
diet alone. Each group of 15 rats was subdivided into three separated groups of
5 males, 5 females, and 5 containing males and females. In the latter group two
litters were born during the first 3 months, but none subsequently. They were
weighed at fortnightly intervals over a year.

The growth curves are shown in Fig. 2. Since no difference of response due
to sex was noted, the average weights refer to all surviving animals. At least
12 animals survived in each group for a year, but the general condition of those
receiving p-toluidine was significantly better than that of the other groups.

393

I

H. G. CRABTREE

The growth-response caused by o-toluidine was negligible, but p-toluidine
acted as a growth-stimulator, particularly in the later stages of the experiment.
The sag in the middle range of the curves is an odd feature for which no explanation
can be offered, but it does not detract from the main result of this experiment.

Observations on these results.

This commentary will be restricted to the experiments in which rats were
used. It makes comparison with previous work easier, since most investigations
have been carried out on rats living under a standardized dietary regime similar
to the one used here, and current hypotheses as to the mode of action of azo-
carcinogens are largely based on such findings.

Though the degree of liver damage and the incidence of liver tumours was
higher in mice than in rats, there is a broad parallelism between the relative
potency of this series of aminoazotoluenes in the two species.

The variable response of different species to a given carcinogen remains a
fundamental problem which cannot, as yet, be expressed in biochemical terms.
Kirby (1945), considering the mechanisms by which the azo-compounds are
degraded in the organism, has suggested that the known alternative paths of
metabolism may vary quantitatively in rats and mice, with formation of benzidine
derivatives (postulated by Cook, Hewitt, Kennaway and Kennaway, 1940) as
the principal reaction occurring in mice, and reductive fission predominating in
rats. On the other ha;nd, Miller and Miller (1947) emphasize the importance of
the protein constitution of the host, and associate the likelihood of tumours
developing with the degree to which an azo-compound is " bound " to the
proteins of rat livers. In the case of rats consuming p-dimethylaminoazobenzene
this fixation is prominent, but does not occur in species resistant to the carcino-
genic action of the substance. These differences can only be attributed to the
special properties of the proteins characteristic for each species.

The " split-product " hypothesis.

Kirby (1945) has reviewed the work bearing on this hypothesis of the
mechanism of carcinogenesis by azo-compounds, and throws doubt on its validity
by citing several examples which fail to conform with its main premises. He
emphasized in particular the low carcinogenic power of p'-methyl-p-dimethyl-
aminoazobenzene when compared with the high activity of the parent p-dimethyl-
aminoazobenzene, since both compounds should yield the same postulated active
diamine split-product. This example, and others to be mentioned, makes it clear
that the other half of the reduced molecule-the amine moiety-plays an impor-
tant role in determining carcinogenic activity if the primary assumption be
accepted that reductive fission is an essential preliminary to this process.

This consideration is emphasized by the results presented here. In fact the
possible significance of the " second half " of the reduced azo-molecule, almost
entirely ignored by previous workers, prompted these experiments. o: o was
known to be carcinogenic, and p: p non-carcinogenic for rats and mice, or
translated in terms of the " split-product " hypothesis, 2: 5-toluylene diamine is
active while 3: 4-toluylene diamine is innocuous.

394'

CARCINOGENIC ACTIVITY OF AMINOAZOTOLUENES

To throw light on the relative contributions made by the amine to diamine
parts of the reduced molecule, the hybrid isomers o p and p: o were prepared
and their action compared with that of the related o o and p: p isomers. The
experiments demonstrated that o: o and o: p were carcinogenic, while p: p
and p: o were non-carcinogenic.

Reviewing these results in the light of the " split-product " hypothesis, no
correlation between carcinogenic activity and the potential diamine fission
products is found. p: p and o: p should yield the same "innocuous " o-toluy-
lene diamine, while o: o and o: p should yield the same "active ".p-toluylene
diamine, but the facts are otherwise, since each of these pairs contains one
carcinogen and one non-carcinogen.

By contrast, when the amine halves of the reduced molecule are considered,
the results show that the two isomers which should yield p-toluidine on reductive
fission-p: p and p: o-are non-carcinogenic, while the pair yielding o-toluidine
-o: o and o p-are carcinogenic. In brief, if carcinogenic activity is indeed
a consequence of the action of the " split-products," it is the amine, and not the
diamine, which determines the subsequent biological response.

The literature contains several examples of the controlling influence which a
methyl group exerts on carcinogenesis, when attached at a para position with
respect to the azo-group. Miller and Baumann (1945) compared the activities
of the o', m', and p' methyl derivatives of p-dimethylaminoazobenzene with that
of the parent compound. The m'-compound was extremely active (7 rats out
of 8 bearing liver tumours in 4 months), the o'-compound moderately active
(4 rats out of 9 bearing tumours in 8 months), and the p'-compound was very
weakly carcinogenic (1 rat out of 10 with a tumour in 10 months). Nagao (1941)
also found that p'-methyl-p-dimethylaminoazobenzene was only a weak carcino-
gen, and that the substances formed by placing a further methyl group in the ring
carrying the dimethylamino-group were entirely innocuous. Sugiura (1948)
tested a series of derivatives of p-methylaminoazobenzene containing an additional
methyl group in the o', m' or p' positions. The order of potency-m'>o'>p'-
was similar to that mentioned above.

All these observations seem relevant to the present work. The common
feature is the great loss of tumour-producing activity which results when a methyl
group is added in the p' position to the parent carcinogenic molecule, forming
derivatives with the potentiality of liberating p-toluidine on reductive fission.

A working hypothesis is put forward tentatively in an attempt to co-ordinate
the data outlined above. When the structural features and the biological action
of these six isomeric azotoluenes are considered, a correlation is evident between
their growth-stimulating properties, their lack of carcinogenic power, and the
presence of an additional methyl group in the position described. The two latter
characteristics are common to all the substances used by Miller and Baumann,
Nagao and Sugiura, but these workers do not mention any differential growth
responses in the rats used in their studies.

The amines emerging from reductive fission may undergo further metabolic
changes (Stevenson, Dobriner and Rhoads, 1942; Elson and Warren, 1944).
They are detoxicated and excreted, in the examples given, as acetylated diamines
or as aminophenols. The breakdown products of the aminoazotoluenes have not
yet been investigated, but presumably the toluidines are initially formed. Jaffe

395

H. G. CRABTREE

and Hilbert (1888) fed the three isomers of acetotoluidine to dogs. o-Aceto-
toluidine was oxidized to an aminophenol and isolated as methylbenzoxalone,
while m- and p-acetotoluidines were excreted as acetylated amino-benzoic acids.
It may therefore be conjectured that all azo-compounds containing a methyl
group at a position para to the azo-group will yield p-aminobenzoic acid as a
final product of their metabolic degradation within the rat.

The experiment pictured in Fig. 2 shows that p-toluidine can stimulate
growth in rats fed on a diet permitting only limited growth and maintenance.
If this is due to the liberation of p-aminobenzoic acid, the question arises: Is the
synthesis of folic acid indirectly influenced by feeding appropriate azo-compounds
via the succession of changes

azo-compound-   > p-toluidine  > p-aminobenzoic acid - > folic acid?

And further, what is the relation of folic acid to the induction of tumours ?

Folic acid, in its relation to the cancer problem, has been widely investigated
in recent years, but its possible association with tumour induction is uncertain,
since the major effort has been made in assessing its therapeutic possibilities.
The early work of Lewisohn, Leuchtenberger, Leuchtenberger and Keresztesy
(1946), who reported regression in a high percentage of transplanted mammary
tumours when pteroyl triglutamic acid was injected intravenously, has been
followed by several clinical trials (Leading Article, 1948). It has also been
shown that the development of Rous sarcoma, after injection of the virus
in baby chicks, was completely prevented by maintaining the birds on a
folic-acid-free diet. Tumour growth was prevented in 40 per cent of birds up
to 6 weeks of age. Certain folic acid antagonists were equally effective for this
purpose, and were also able to cause cessation of growth in Rous sarcomas already
established (Little, Sampath, Paganelli, Locke, and Subbarow, 1948).

In the field of bacterial metabolism there is strong evidence that p-amino-
benzoic acid is concerned with the synthesis of folic acid, purines, thymine and
methionine, and that the sulphlonamides may inhibit reactions leading to the
synthesis of proteins and nucleic acids (Woods, 1947).

The folic acid requirements of rats have not been studied so widely as those
of chicks, since the symptoms of deficiency are not so easily recognized. Woolley
(1947) states that " experimental tricks such as the administration of drugs are
necessary to produce a dietary need for the vitamin." This is exemplified by the
haematological response following the- administration of sulphonamides to rats
which is reversed by injection of folic acid (Endicott, Daft and Ott, 1945), or the
similar changes produced by protein depletion or inadequate riboflavine (Korn-
berg, Daft and Sebrell, 1945).

It is tempting to interpret the experimental results described in this paper in
terms of folic acid balance. The diamines resulting from the reductive fission
of azo-carcinogens have been shown to inhibit many enzyme reactions under
oxidative conditions, and it is conceivable that they interfere with the function
of enzymes concerned in the synthesis of folic acid, producing adverse effects
comparable to those produced by sulphonamides, though by a different mechanism.
Conversely the non-carcinogenic, growth-stimulating isomers of amino-azo-
toluene could be visualized as precursors of p-aminobenzoic acid, the liberation
of which could counteract the damage produced by depletion of folic acid.

396

CARCINOGENIC ACTIViTY OF AMINOAZOTOLUENES            397

The limited growth conditioned by a diet low in proteins and inadequate in
its complement of B-vitamins reinforced by the postulated inhibition of folic
acid synthesis would then constitute the metabolic distutbances favourable for
the emergence of tumours.

SUMMARY.

1. Six isomeric aminoazotoluenes have been tested for their carcinogenic
activity in the livers of mice and rats, when added at 0 06 per cent concentration
to a standard, semi-synthetic diet. They are labelled o: o, m: m, p: p, o: p,
p: o and p: m respectively to denote the two toluidines used in their preparation.

2. A series of characteristic histological changes, reflecting increasing degrees
of liver damage, has been used in classifying the results, which serve to indicate
the relative potency of the six isomers.

3. Mice were more susceptible than rats, though the order of potency, with one
exception, was the same in both species. All the isomers, except p: p, proved
carcinogenic for mice, but only two of them-o: o and o: p-were carcinogenic
for rats.

4. The growth-curves of each group of rats consuming the different amino-
azotoluenes are shown. The two carcinogens, o: o and o: p, did not influence
the growth rate, but the four non-carcinogens-p: o, p: p, m: m and p: m-
stimulated growth in varying degrees the p: p isomer being the most effective.

5. Rats were fed on the same basal diet containing 0 06 per cent of either
o- or p-toluidine. o-Toluidine produced no effect on growth, but p-toluidine
acted as a growth stimulant.

6. These results are discussed in the light of the " split-product " hypothesis,
which they fail to support. Also, on the assumption that carcinogenic properties
and growth effects are conditioned by their " split-products," a correlation had
been established between growth stimulation, non-carcinogenicity and the release
of p-toluidine.

7. Since the detoxication of p-toluidine can produce p-aminobenzoic acid,
the possible relation between folic acid metabolism and the mechanism of cancer
induction is considered.

I am greatly indebted to Dr. E. Vazquez Lopez for help and guidance in the
histological aspects of this work. The method of classification shown in Table I
was suggested by him, and, in assessing the degree of liver damage, his judgment
was final.

REFERENCES.

COOK, J. W., HEWITT, C. L., KENNAWAY, E. L., AND KENNAWAY, N. M.-(1940) Amer.

J. Cancer, 40, 62.

CRABTREE, H. G.-(1947) Brit. med. Bull., 4, 345.

ELSON, L. A., AND HOCH-IiGETI, C.-(1944) Biochem. J. (Proceedings), 38, x.
Idem AND WARREN, F. L.- (1944) Ibid., 38, 217.

ENDICOTT, K. M., DAFT, F. S., AND OTT, M.-(1945) Arch. Path., 40, 364.
FISCHER, B., AND WIMMER, H.-(1887) Berichte, 20, 1581.

HARIS, P. N., KRAHL, M. E., AND CLOWES, G. H. A.-(1947) Cancer Res., 7, 162.
JAFFE, M., AND HILBERT, P.-(1888) Z. Physiol. Chem., 12, 295.

KENSLER, C. J., DEXTER, S. O., AND RHOADS, C. P.-(1942) Cancer Res., 2, 1.
KIRBY, A. H. M.-(1945) Ibid., 5, 683.-(1947) Ibid., 7, 333.

398                      S. BRUZZONE

KORNBERG, A., DAFT, F. S., AND SEBRELL, W. H. (1945) Arch. Biochem., 8, 431.
Leading article, (1948) Brit. Med. J., ii, 827.

LEwISONm, R., LEUCHTENBERGER, C., LEUCHTENBERGER, R., AND KERESZTESY, J. C.

-(1946) Science, 104, 436.

LiTTLE, P. A., SAMPATH, A., PAGANELLI, V., LOCKE, E., and SUBBAROW, Y.-(1948)

Trans. N.Y. Acad. Sci. 10, 91.

MEHNER, H.-(1902) J. prakt.( Chem., 65, 401.

MILLER, E. C., AND MILLER, J. A.-(1947) Cancer Res., 7, 468.
MILLER, J. A., AND BAUMANN, C. A.-(1945) Ibid., 5, 227.

Idem, MINER, D. L., RUSCH, H. P., AND BAUMANN, C. A.-(1941) Ibid., 1, 699.
NAGAO, N. (1941) Oann, 35, 8.

NIETzKI, R.-(1877) Berichte, 10, 662.

POTTER, V. R.-(1942) Cancer Res., 2, 688.

STEVENSON, E. S., DOBRINER, K., AND RHOADS, C. P.-(1942) Ibid., 2, 160.
SUGIURA, K.-(1948) Ibid., 8, 141.

WOODS, D. DI-(1947) Ann. Rev. Biochem., 16, 613.
WOOLLEY, D. W.-(1947) Ibid., 16, 372.

ZINCKE, T., AND LAWSON, A. T.-(1886) Berichte, 19, 1452.

				


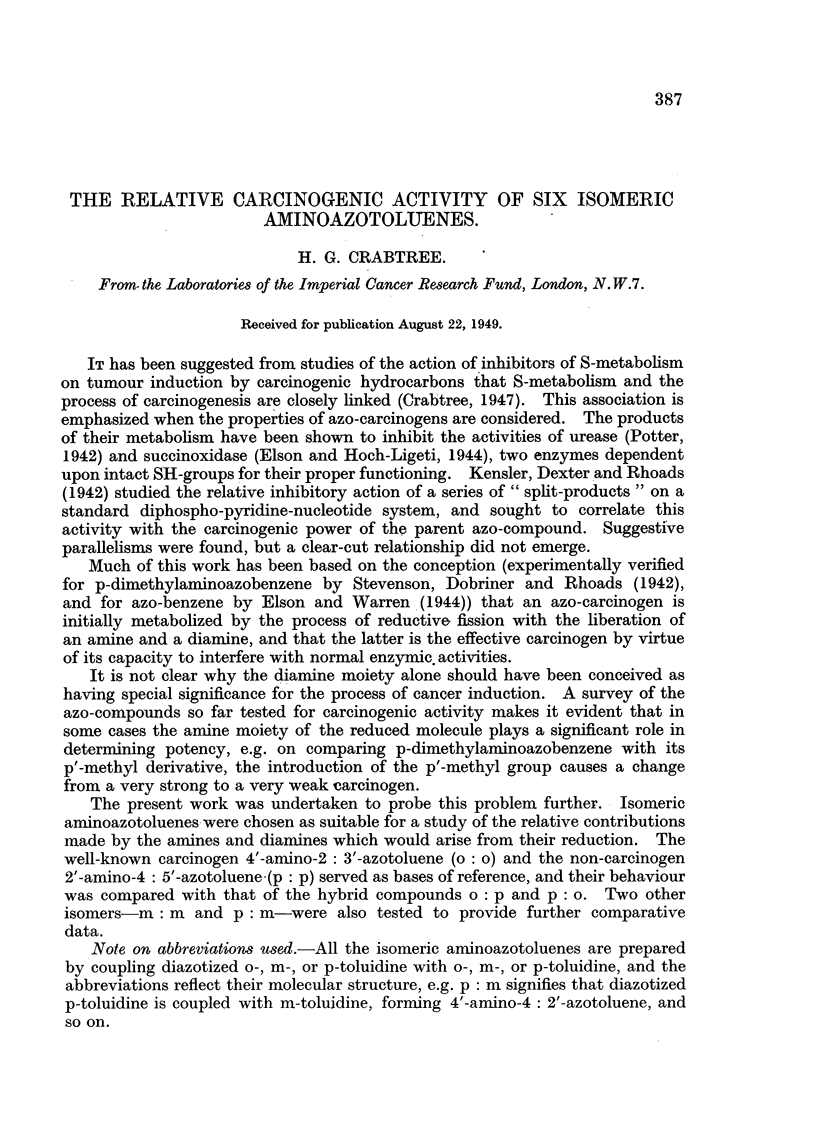

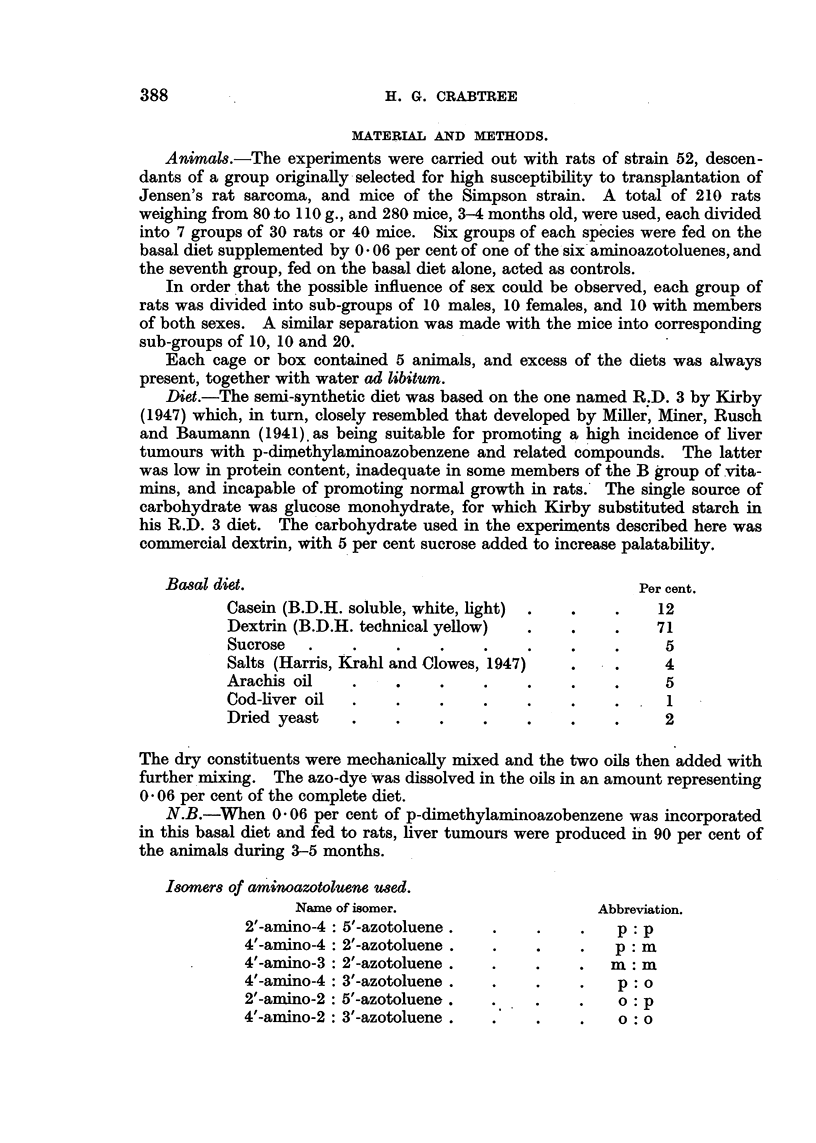

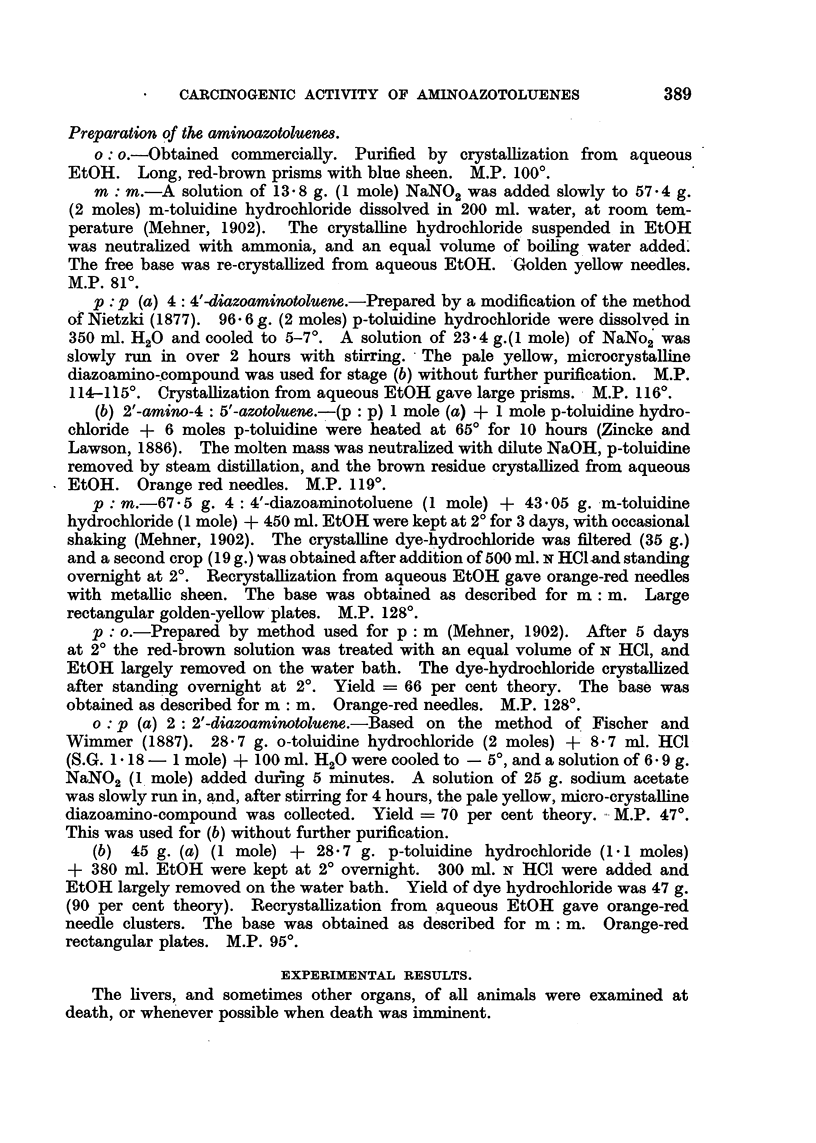

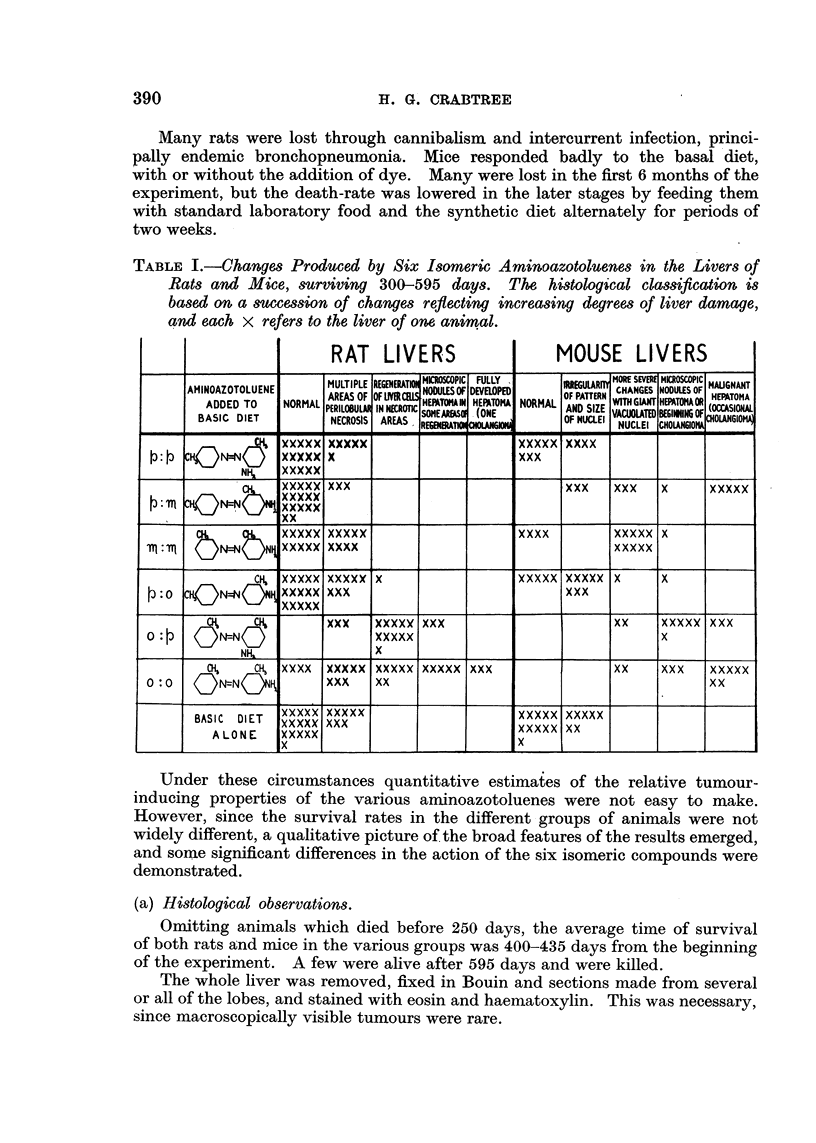

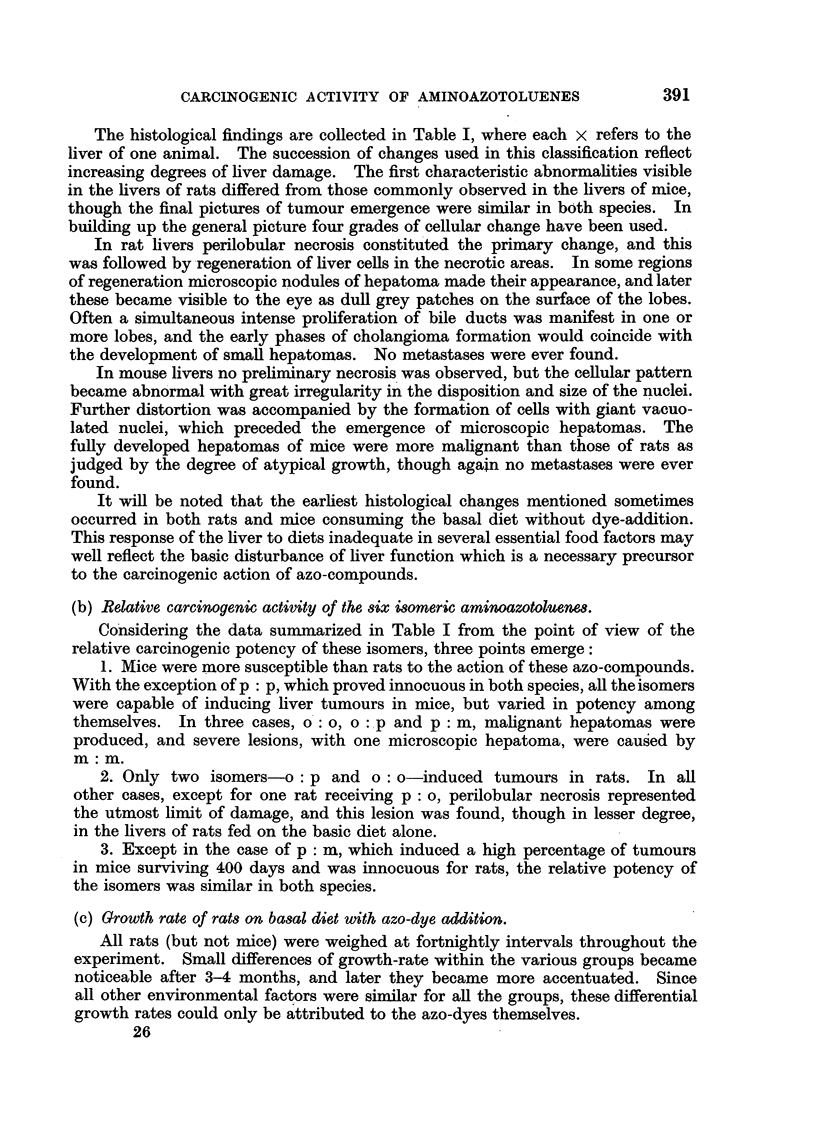

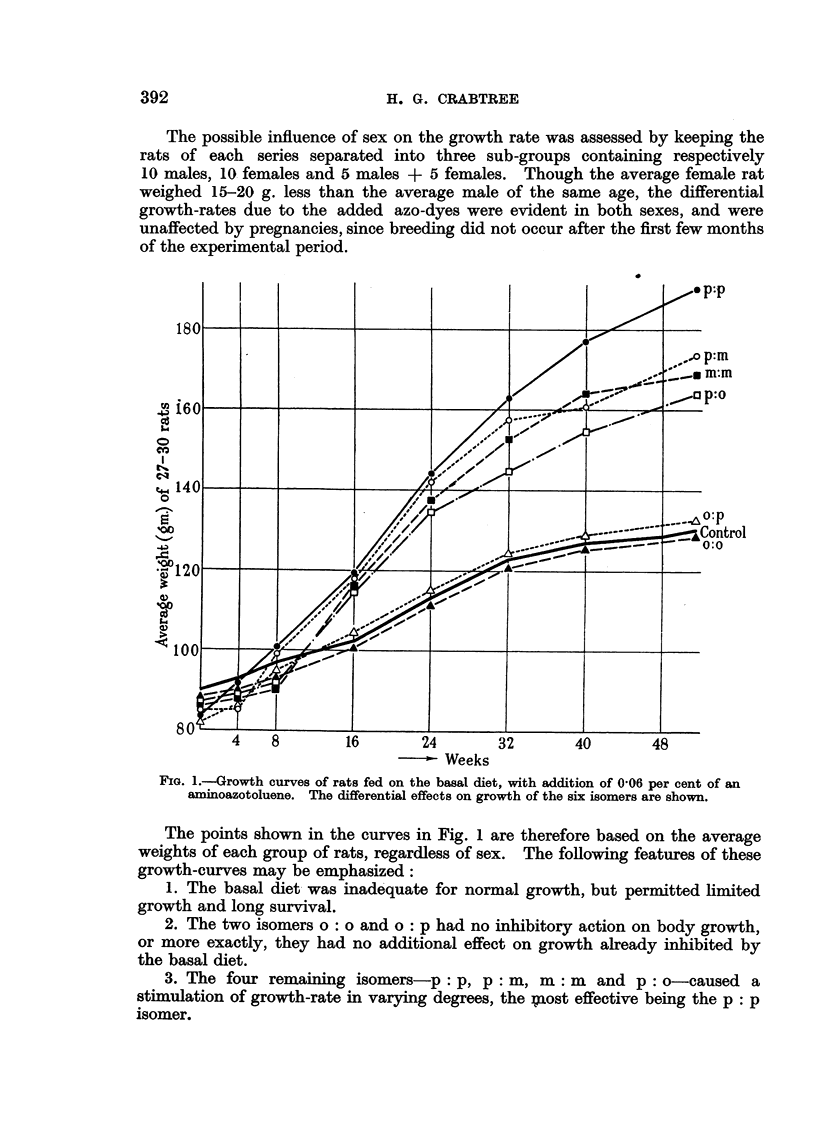

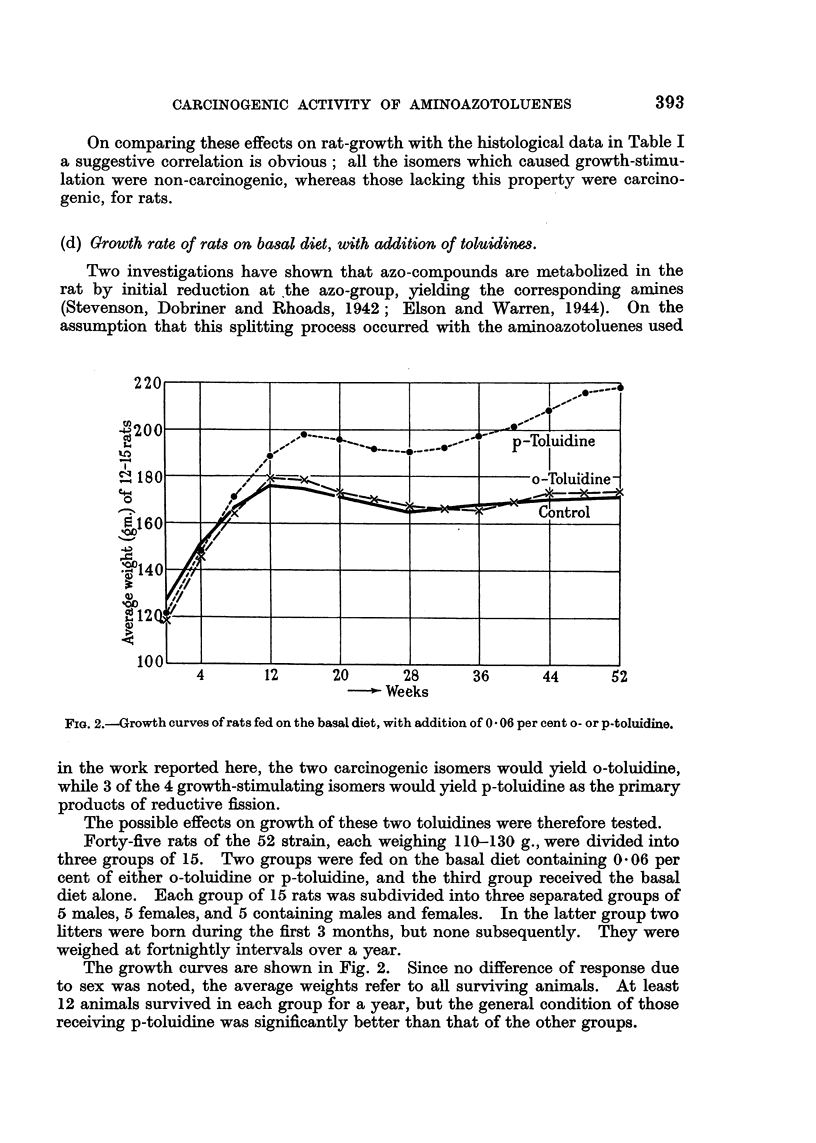

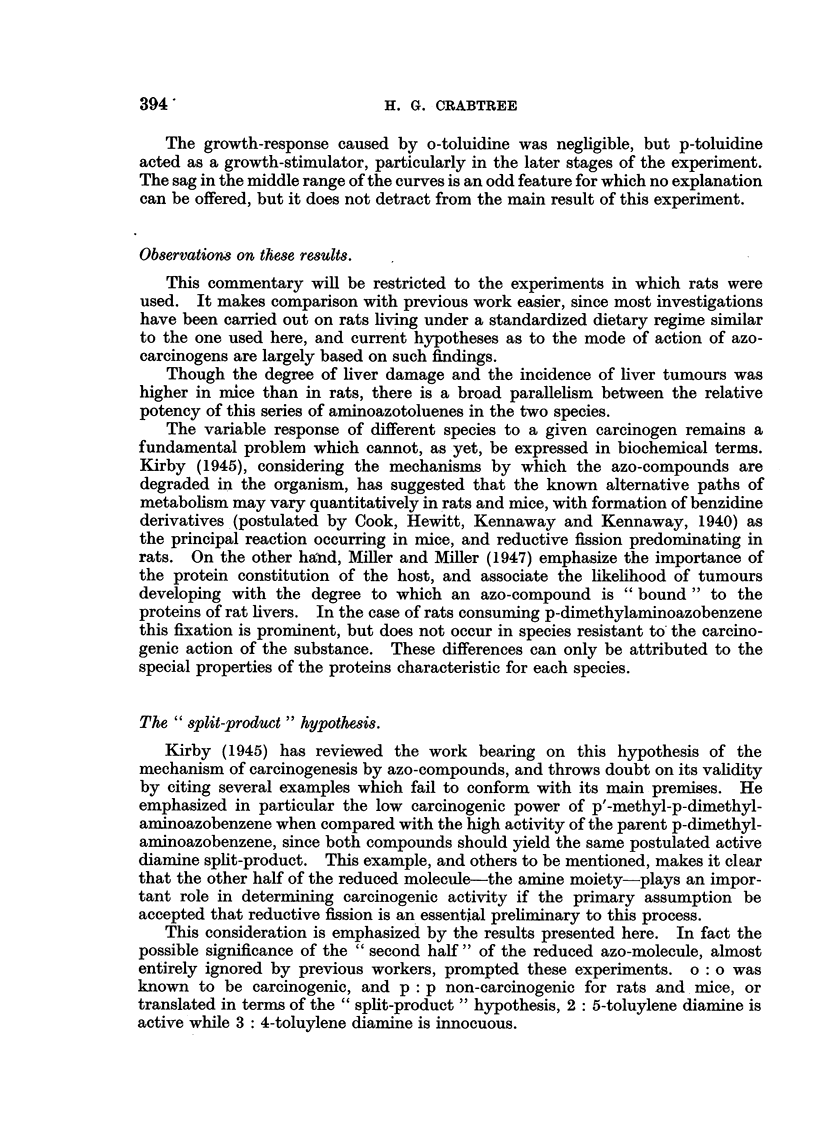

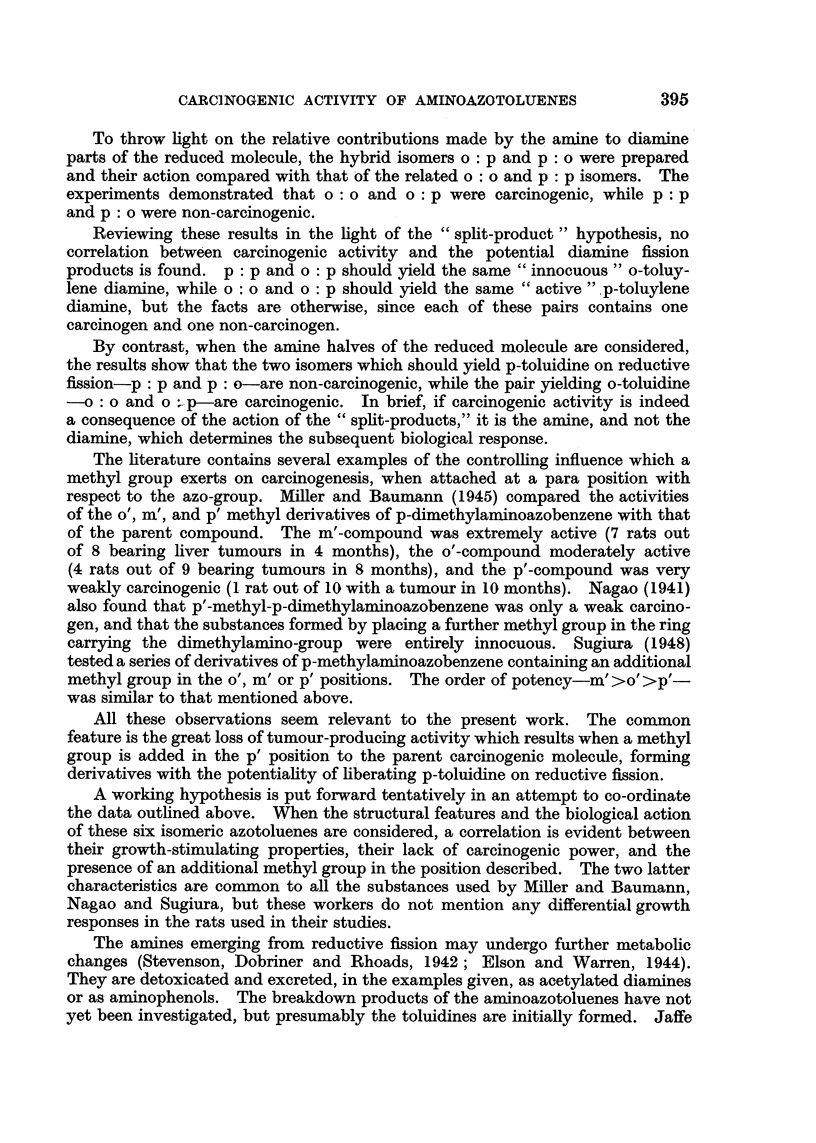

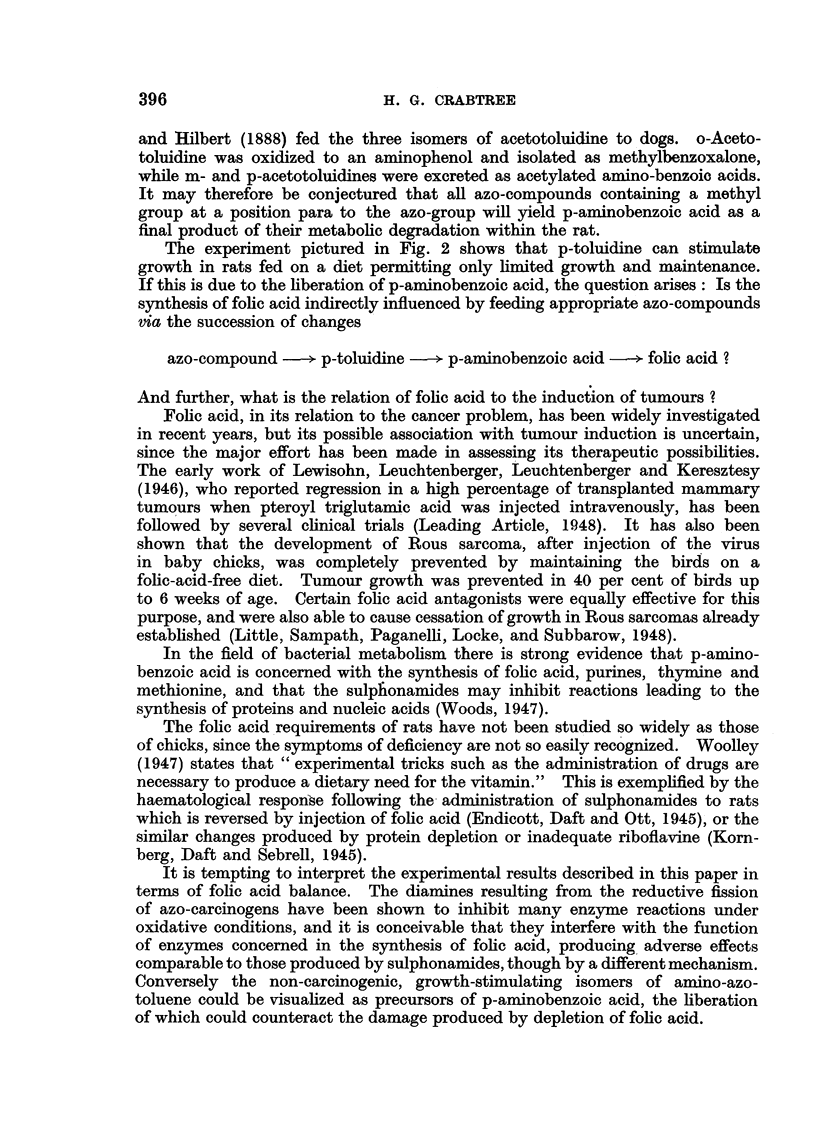

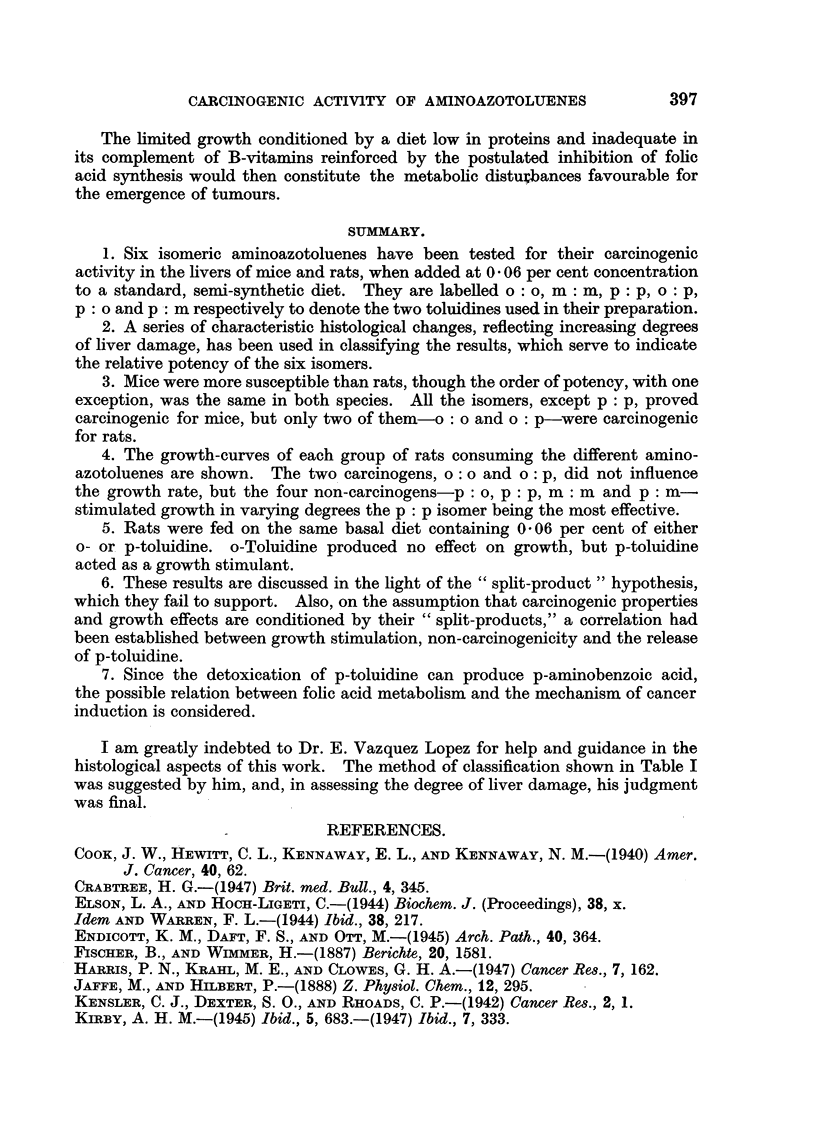

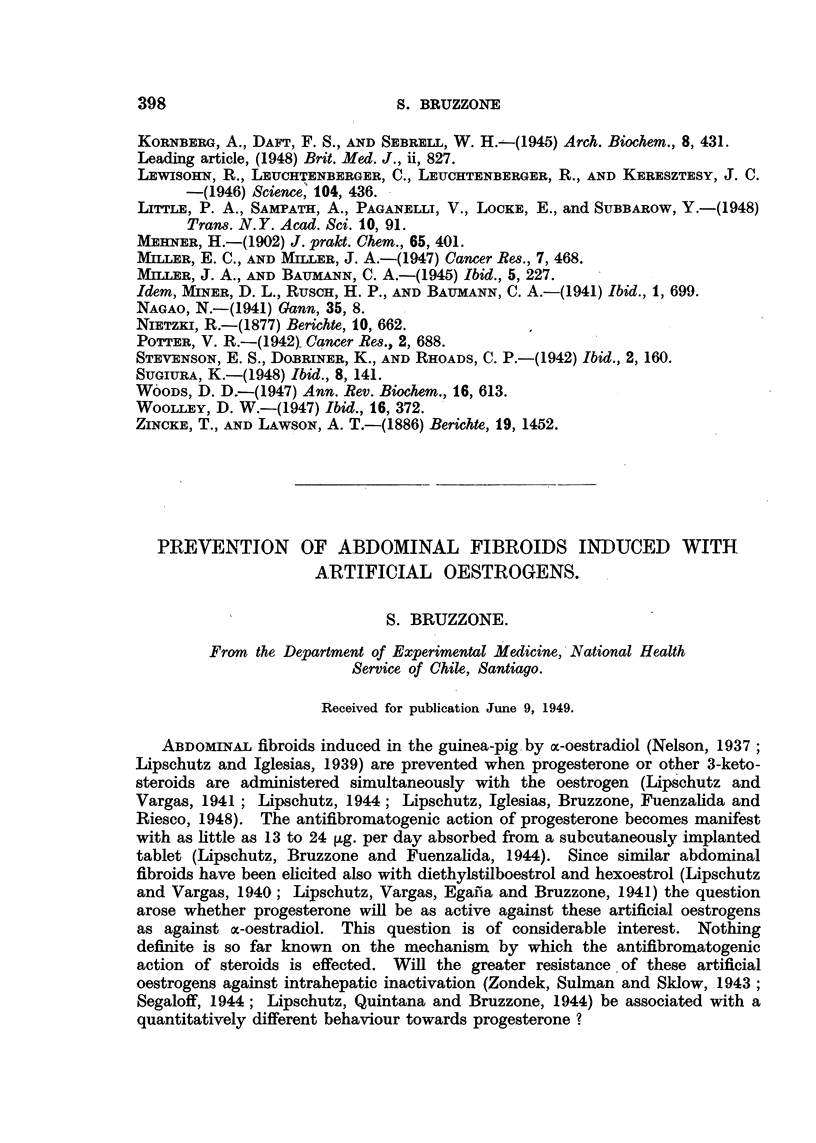

